# Protocols for management of oral complications of chemotherapy and/or radiotherapy for oral cancer: Systematic review and meta-analysis current

**DOI:** 10.4317/medoral.21314

**Published:** 2016-12-06

**Authors:** José-Nunes Carneiro-Neto, Juliana-Dreyer-da-Silva de-Menezes, Lucas-Borin Moura, Elaine-Maria-Sgavioli Massucato, Cleverton-Roberto de-Andrade

**Affiliations:** 1 Master Student in Dental Science, Diagnostics and Surgery Area, Araraquara Dental School, UNESP - Univ Estadual Paulista, Araraquara, Sao Paulo, Brazil; 2 Specialist in Surgery and Traumatology Buco-Maxillofacial by APCD Bauru and the Base Hospital. Master in Rehabilitation Sciences at HRAC-USP. Doctoral Student in Dental Science, Diagnostics and Surgery Area, Araraquara Dental School, UNESP - Univ Estadual Paulista, Araraquara, Sao Paulo, Brazil; 3 Specialist in Surgery and Traumatology Buco-Maxillofacial by Hospital School of the Federal University of Pelotas. Doctoral Student in Dental Science, Diagnostics and Surgery Area, Araraquara Dental School, UNESP - Univ Estadual Paulista, Araraquara, Sao Paulo, Brazil; 4 Master and Doctor of Dentistry, Stomatology Area, Araçatuba Dental School, UNESP. Director and Assistant Professor, Department of Diagnostics and Sugery, Araraquara Dental School, UNESP - Univ Estadual Paulista, Araraquara, Sao Paulo, Brazil; 5 Master in Biopathology Buccodental, Doctor of Stomatology, University of Campinas, Unicamp. Assistant Professor and Pathologist, Department of Physiology and Pathology, Araraquara Dental School, UNESP - Univ Estadual Paulista, Araraquara, Sao Paulo, Brazil

## Abstract

**Background and Objectives:**

Considering the high possibility of dentist consult a patient with oral complications of chemotherapy and/or radiotherapy for oral cancer because of the advances in this area, this study aims to systematically review the literature to identify and suggest effective and safe protocols for the managements of oral complications in oncology patients.

**Material and Methods:**

TThe systematic review was designed by PICO and PRISMA including eligibility and exclusion criteria; the source of information and search strategy in PubMed according MeSH: “Mouth Neoplasms and Radiotherapy” and “Mouth Neoplasms and Drug Therapy” the period from 2010 to 2015; selection and data collection of study was carried form blind and independently by two researchers; risk of bias and methodological quality: ensured by the PEDro scale; synthesis of data: of oral complications were evaluated by adapted version of associative direction classification proposed by Costigan and collaborators; and data analysis was performed by the meta-analysis of BioEstat program (5.0) in the included studies.

**Results:**

2,700 articles found, 2,371 were selected after removal of duplicate and elected 40 full-text articles. Of these, only 06 articles were included in the systematic review with exclusion of others, per obtain punctuation ≥ 7 with high methodological quality for synthesis of the managements of oral complications. Since 05 articles were associated with low risk of bias composing the protocols suggestive for managements and the meta-analysis in odds ratio (0.916) to cure and relative risk (1.049) for the development of oral mucositis and pain.

**Conclusions:**

The protocols suggestive for managements of oral mucositis and pain with MuGard - mucoadhesive hydrogel; PerioAid Tratamiento® antiseptic mouthrinse with chlorhexidine and cetylpyridinium chloride; Episil® plus benzydamine - bioadhesive oromucosal gel; 0,03% of Triclosan mouthwash Colgate Plax; and Diode Laser Therapy of low-level are safe for oncology patients applied according to adopted clinical parameters.

**Key words:**Oral cancer, radiotherapy, chemotherapy,complications, management.

## Introduction

Despite the recent increase in the incidence of Oral Squamous Cell Carcinoma (OSCC) in younger patients (1) and of gender female (2), yet the prevalence is in older males (2) between the 5th and 8th decade of life associated with high consumption of alcohol and tobacco. Since 4% of all oral malignancies are found in patients less than 40 years (3).

The OSCC is the most common malignancy of the oral cavity with high lethality when diagnosed in tongue and floor in advanced stages (3,4), representing 263,000 new cases and 127,700 deaths worldwide/year (2). The main modalities of contemporary treatments for oral cancer include surgical resection, chemotherapy (CT), radiotherapy (RT) and transplant immunotherapy of hematopoietic stem cells isolated or in combination (5). The modalities RT, CT and/or chemoradiotherapy (CRT) have a high potential of produce direct damage to tissues of the oral cavity or production of haematopoietic cells, because these have a high rate of cell turnover between 7 to 14 days (6). These oral complications are referred to as oral mucositis, dysgeusia, infectious diseases (5) and xerostomia associated with loss of glandular function. Together, these oral complications are called Oral Complications of Chemotherapy and/or Radiotherapy for Oral Cancer (OCCROC) (4). It is clear that high prevalence of oral cancer associated with advances in detection and application of new treatment modalities increase the possibility of the dentist come across, in his dental office, with patients presenting clinical condition of OCCROC (7). Thereby, we consider important to organize a study of systematic review of the topic in order for systematize and organize published data considering high methodological quality and low risk of bias and so identify and suggest effective and safe protocols for the managements of OCCROC in oncology patients.

## Material and Methods

The systematic review was developed according to the recommendations of the PRISMA (8), except for protocol and registration, delimited you for the following PICO (patient, intervention, comparison and objective): “In patients with OCCROC, current protocols for management of the experimental group (EG) are safer and more effective than the control group (CG) to reduce/cure these oral complications?”.

- Eligibility criteria

The inclusion criteria for the selection of articles were: [1] pattern of publication in a journal or magazine with full text online through a Virtual Private Network of Paulista State University; [2] search time of publication 01/01/2010 to 31/05/2015; [3] target population of all age groups with staging of oral cancer I-IV; [4] oncological treatment with any dose Gray (Gy) to radiotherapy and/or chemotherapy medication; [5] management comparison of EG and CG for OCCROC; [6] Language English; and [7] design study of clinical trial. We excluded articles that did not attend the inclusion criteria.

- Information sources and search strategy

By means of research conducted in electronic databases of PubMed, with the following descriptor in English according to the MeSH (Medical Subject Headings): “Mouth Neoplasms” combined with “and” and qualifiers individually “Radiotherapy” and “Drug Therapy”, in the period 11 to 15 May 2015.

- Study selection, collection process and data items

Performed independently and blindly by two researchers/authors (X and Y) respecting the criteria for inclusion and exclusion previously mentioned. Specifically, was performed to identify the search strategy of the articles using titles, abstract and full text, collecting the following relevant information according to the objective of the research: primary author and year, country, study design, target population, oncological treatment, oral complications, managements, follow-up and effectiveness (classified according to three simple rule to the results of EG and CG, as poor of 0-16%, bad of 17-33%, regular of 34-49%, good of 50-66%, optimum of 67-82% and excellent of 83-100%).

- Risk of bias and methodological quality

The quality of the information collected in each selected article for the production of systematic literature review and protocols suggestive for managements of the thematic in question was ensured through the standardization of PEDro Scale, due to their effectiveness and practicability in the article validation (9).

The criteria of the PEDro Scale were originally developed to be used in experimental studies in humans, standardized on a 0-10 point score for quality of scientific evidence, with punctuation for the following criteria: 1) specification of the inclusion criteria (item not punctuated); 2) randomized allocation; 3) confidentiality allocation; 4) similarity of groups at baseline or the basal phase; 5) blinding subjects; 6) blinding therapist; 7) blinding evaluator; 8) measure of at least one primary outcome in 85% of subjects allocated; 9) analysis of intention to treat; 10) comparison between groups of at least one primary outcome and 11) reporting variability measures and estimation of the parameters of at least one primary variable (9).

For the criteria of exclusion and inclusion examiners proceeded the following rule: excluded from the review the articles with low [≤ 3 points] and moderate [4-6 points] methodological quality high and moderate risk of bias, respectively, including the others [≥ 7 points] of methodological quality high and low risk of bias in the systematic review.

- Synthesis of data

Regarding the association of OCCROC with the management of EG, the studies included in the systematic review were evaluated by adapted version of associative direction classification proposed by Costigan *et al.* (10), through the association [positive, negative or no] of the outcome of management of EG with the indicator of oral complications. Pursuing with the codification of the results between studies equally associated by three simple rule of 0-33% as “0” (association without), of 34-59% as “?” (indeterminate association) and of 60-100% as “+” (positive association) or “–” (negative association). And posteriorly with the codification of the low risk of bias between four/more studies equally associated with the same previous parameter represented by “0 0” (association without), “? ?” (indeterminate association) and “+ +” (positive association) or “– –” (negative association) (11). This last quality of the studies combined to score 7-10 points of the PEDro Scale protocols suggestive for managements of patients with OCCROC.

- Data analysis

The meta-analysis was performed in BioEstat program (5.0) combining the data of the managements of EG and CG for oral mucositis and pain (OMP), considering the final values of degree and intensity OMP obtained in the clinical trials presented in the suggested protocols. The degree of homogeneity or heterogeneity of the studies was evaluated by statistical test Chi-square (χ2) resulting in the acceptance of the null hypothesis (H0) in both tests, due the value of *P* = 0.8985 (homogeneity) and *P* = 0,5299 (heterogeneity) be ≥ to decision level α=0.01 and α=0.05, respectively. The H0 of both tests applied to studies of this review confirm that the samples are heterogeneous, in this case, is indicated by the data analysis Random Effects Model of DerSimonian-Laird (12) for evaluation of the following Odds Ratio tests (OR) together with the Relative Risk (RR) to quantify the association between the presence of OMP and the effectiveness of managements of EG and CG with confidence interval of 95% (CI 95%) and decision value of OR (*P* < 0.05) and RR (*P* < 0.01) for statistical significance (13-15).

## Results

1- Overview of studies

**Figure 1:**
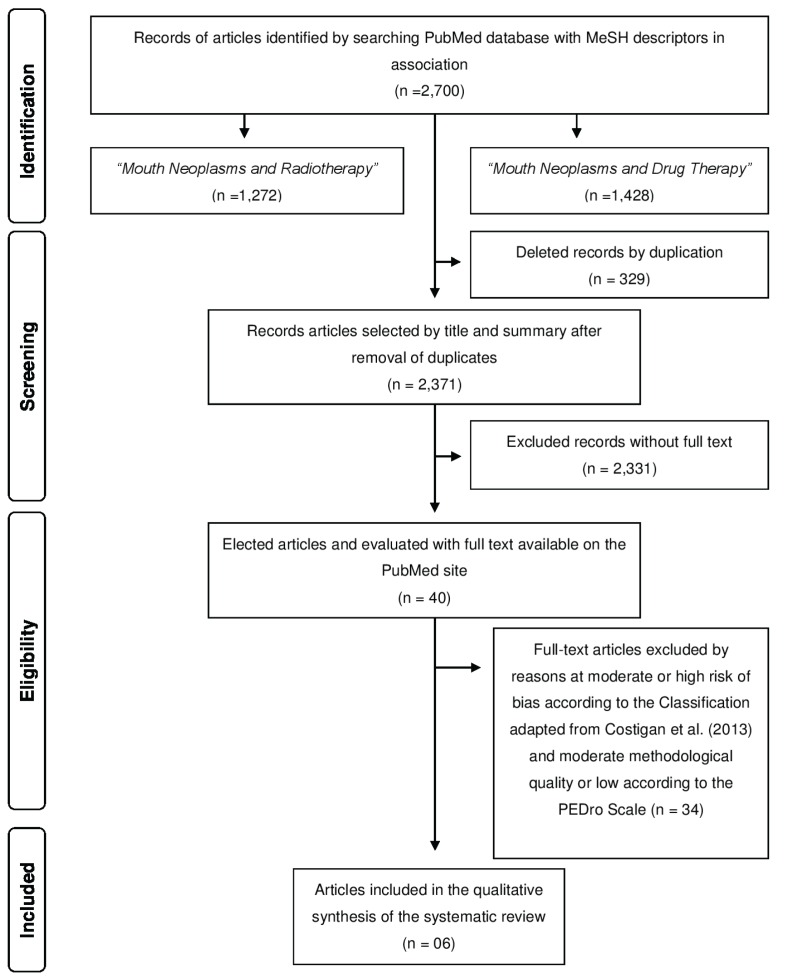
Flow chart of search strateggy and selection of the articles included in the systematic review in accordance with the PRISMA (8)

Were found, according to the eligibility criteria and search strategy of the articles in electronic databases of PubMed in the period from 11 to 15/05/2015, a total of 2,700 articles using three descriptors of the MeSH in the following association, “Mouth Neoplasms and Radiotherapy” and “Mouth Neoplasms and Drug Therapy”, of which we selected 2,371, removing duplicate articles. 40 articles were elected containing full text available at PubMed for qualification methodology and assessment of risk of bias by PEDro Scale. Only 06 articles obtained punctuation ≥ 7 with high methodological quality, criteria considered necessary for inclusion this systematic review (Fig. 1 and  Table 1 and 1 continue ).

**Table 1:**
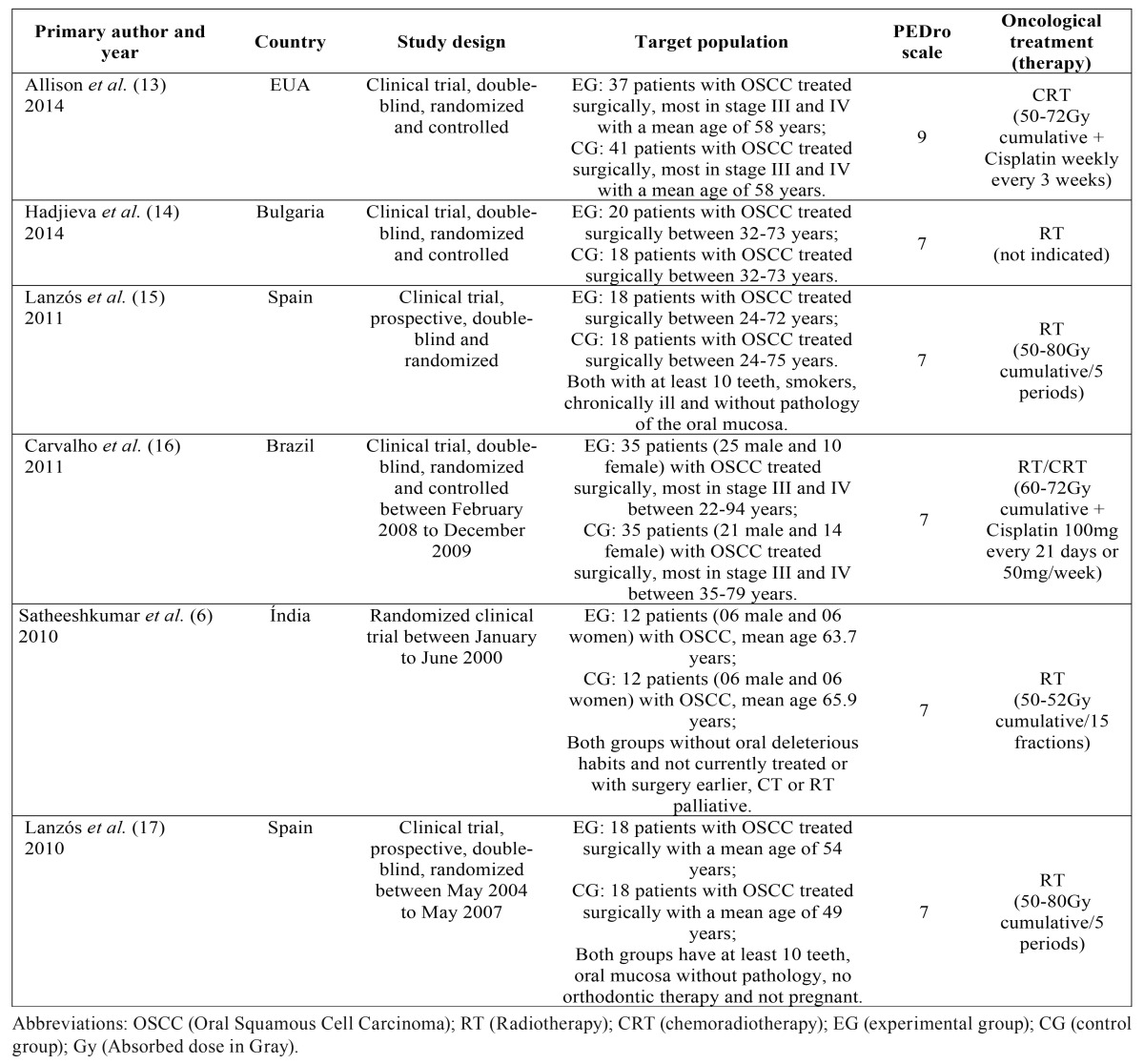
Characteristics of the studies included in the systematic review (Continue).

**Table 1 continue:**
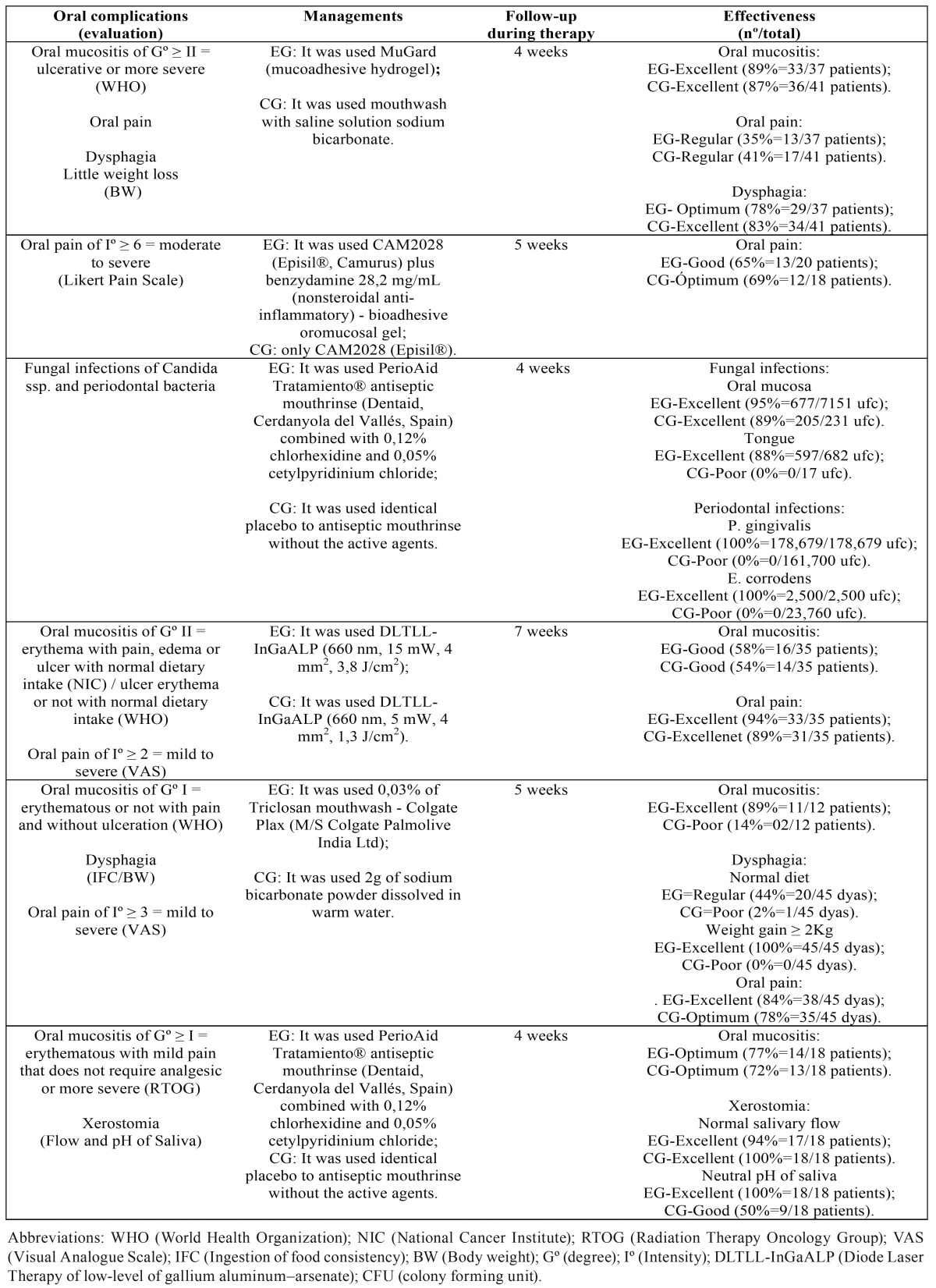
Characteristics of the studies included in the systematic review (Conclusión).

Of the 06 articles included, five articles obtained punctuation 7 corresponding to RT treatment and an article obtained punctuation 9 related to the treatments CRT, respectively ( Table 1and 1 continue ). These articles were submitted to the adapted version of associative direction classification (10), result of the following associations, in its most positive, between the index of oral complications and the management of EG, but with little positive coding the results of studies and low risk of bias ( Table 2).

**Table 2:**
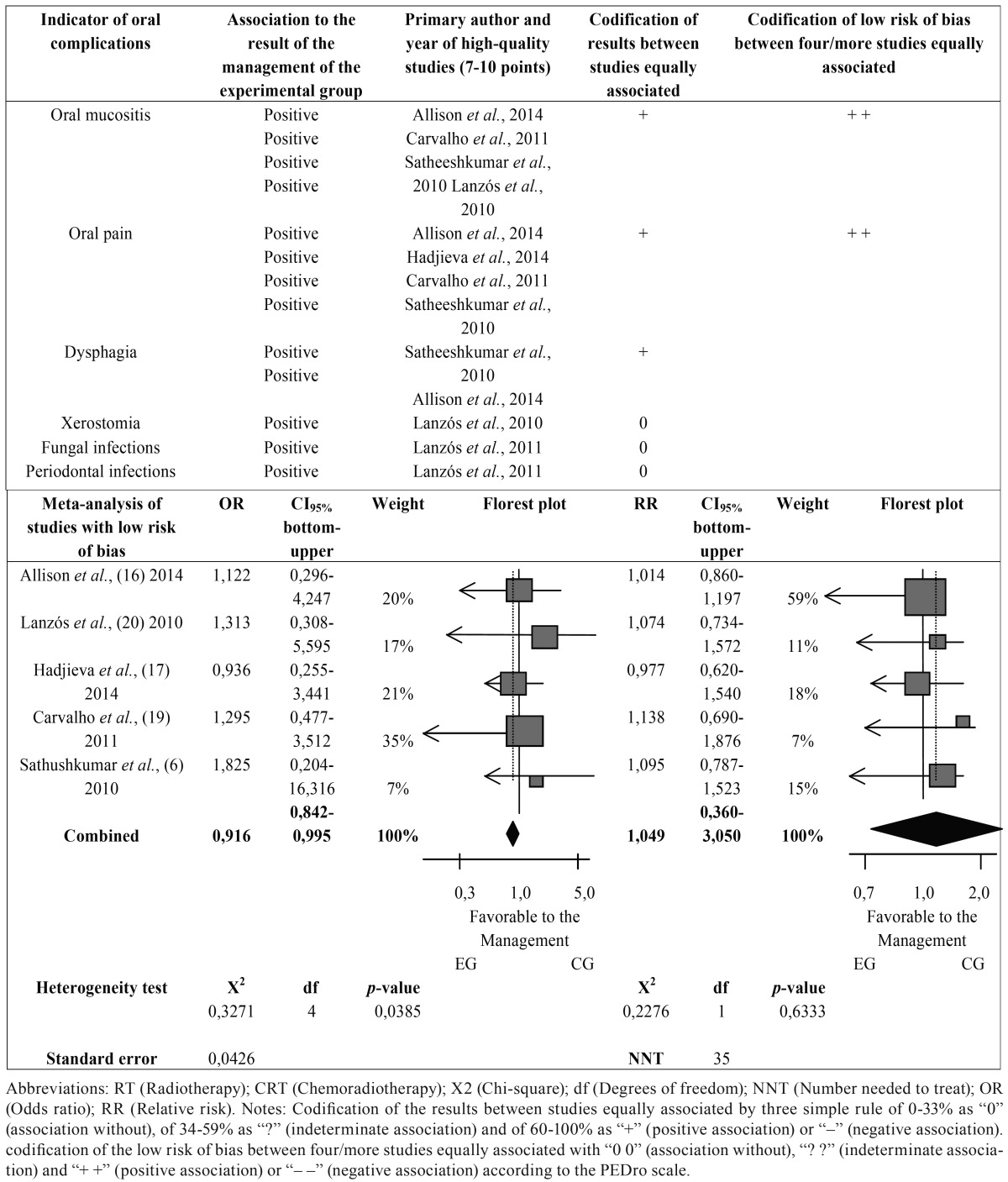
Classification of the studies included in the systematic review with high methodological quality followed by meta-analysis of studies with low risk of bias.

2- Risk of bias

The punctuation total of 40 selected articles varies between 4 and 9, moderate to low risk of bias. Specifically, 20 articles presented punctuation 4 (RT=11, CT=02 and CRT=07), 06 articles punctuation 5 (RT=01, CT=01 and CRT=04) and 08 articles punctuation 6 (RT=03, CT=01 and CRT=04).

The 06 articles identified with low risk of bias demonstrated study design as clinical trial, prospective, retrospective, double-blind, randomized or controlled. And according to the direction associative classifications 05 articles were associated (+ +) with low risk of bias in only two indicators of oral complications so composing the protocols suggestive for managements of OCCROC ( Table 3).

3- Synthesis of the managements of OCCROC 

3.1- Oral mucositis

Of the four studies, all positively evaluated the association of oral mucositis with managements of EG with punctuation between 7 and 9 in the PEDro Scale (clinical trial, prospective, retrospective, double-blind, randomized or controlled). The results coding of 80% (+) were checked in the reduction of the degree of oral mucositis as indicated in the protocol suggested in table 3 through managements with MuGard - mucoadhesive hydrogel (13), Diode Laser Therapy of low-level of InGaAlP (16), 0,03% of Triclosan mouthwash - Colgate Plax (6) and PerioAid Tratamiento® antiseptic mouthrinse combined with 0,12% of chlorhexidine and 0,05% of cetylpyridinium chloride (17).

3.2- Oral pain

Of the four studies, all positively evaluated the association of oral mucositis with managements of EG with punctuation between 7 and 9 in the PEDro Scale (clinical trial, retrospective, double-blind, randomized or controlled). The codification of studies was 80% (+) with positive results in the reducing of the intensity of oral pain as indicated by the protocol suggested in  Table 3 through managements with MuGard - mucoadhesive hydrogel (13), CAM2028 - Episil® plus benzydamine - bioadhesive oromucosal gel (14), Diode Laser Therapy of low-level of InGaAlP (16) and 0,03% of Triclosan mouthwash - Colgate Plax (6).

**Table 3:**
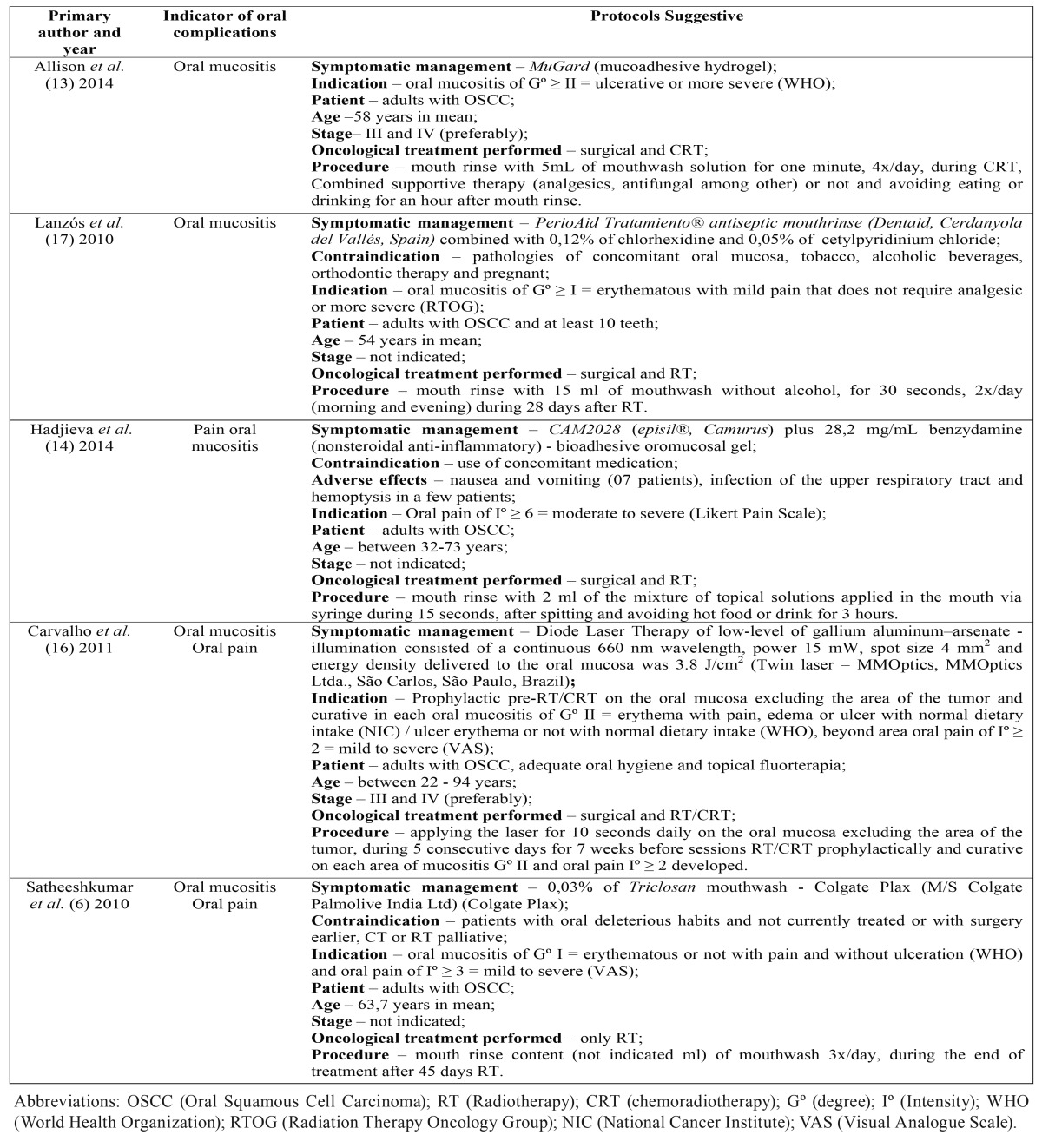
Distribution of protocols suggestive for management of oral complications of chemotherapy and / or radiotherapy for oral cancer.

3.3- Dysphagia

Two studies with 7 and 9 points on the PEDro scale with statistical designs of clinical trial, double-blind, randomized or controlled, presented a positive association in control of dysphagia with managements of EG, coding 100% of your results (+) using the MuGard - mucoadhesive hydrogel (13) and 0,03% of Triclosan mouthwash - Colgate Plax (6) according to the protocol suggested in  Table 3.

3.4-Xerostomia

A study with 0% coding result (0) association without, punctuation 7 on the PEDro scale was related positively the association between xerostomia and the use of PerioAid Tratamiento® antiseptic mouthrinse combined with 0,12% of chlorhexidine and 0,05% of cetylpyridinium chloride (17) according to the protocol suggested in table 3.

3.5- Periodontal infections and fungal

Each oral compilation indicator is not coded by presenting only one study with the result of 0% (0). However, the study obtained scores 7 points on the PEDro Scale (clinical trial, double-blind, randomized and prospective). Associating yourself positively using PerioAid Tratamiento® antiseptic mouthrinse combined with 0,12% of chlorhexidine and 0,05% of cetylpyridinium chloride, in the reduction of Candida spp. colonies in the oral mucosa and tongue, as well as in samples of subgingival periodontal pathogens (P. gingivalis e E. corrodens) during the RT (15) according to the protocol suggested in table 3.

4- Meta-analysis

Only five studies were qualified and analyzed with a total sample of 246 patients. We use effectiveness rates of the managements of EG and CG for OMP coming from the QT and/or RT OSCC (Table 1 and 1 continue). The OR combined demonstrated 0.916, CI 95% = 0.842 - 0.995, weight of 10.922, χ2 = 0.3271, df = 4, *p*-value = 0.0385 and standard error of 0.0426, resulting in statistically significant in the acceptance of the alternative hypothesis (H1) due the *p* value of 0.0385 be ≤ to α = 0.05. The H1 confirming a higher proportion of effectiveness rates of the management of EG for odds ratio in healing the OMP, demonstrating 0.9 times greater therapeutic efficacy of the studies (13,14,16) compared to their CG and managements of the studies (6,17) represented by the diamond in the forest plot ( Table 2).

The RR combined showed 1.049, CI 95% = 0.360 - 3.050, weight of 236.094, χ2 = 0.2276, df = 1, *p*-value = 0.6333 resulting in the acceptance of the null hypothesis (H0) without statistical significance, due the *p*-value of 0.6333 be ≥ to α = 0.01. The H0 confirming a relative risk of OMP in individuals of both managements of the studies (6,13,16,17), showing 1.05 times greater risk of developing of OMP in the management of CG of the studies (6,13,17) represented by the diamond in the forest plot compared to management of CG of the study (16) and both managements of the study (14), being that the group of 35 patients is the number needed to treat (NNT) in the set of management of CG of the studies (6,13,16,17) amenable to occur an OMP ( Table 2).

## Discussion

- Summary of evidence and limitations

The most of the studies of high methodological quality and Low risk of bias according to PEDro Scale (9) and the associative direction classification (10) were developed in Spain. The indicators of oral complications emanated from oncological patients surgically treated for oral cancer during RT were: oral mucositis, oral pain, oral dysphagia, xerostomia, periodontal infections and fungal; after RT: oral mucositis, oral pain, oral dysphagia and xerostomia; during and after CRT: oral mucositis, oral pain, oral dysphagia and xerostomia. Being that the mucositis, pain and oral dysphagia were emanated only to oncological patients during and after RT as a single treatment. Regarding protocols of the managements of the OCCROC the age of the patients varied between 22 to 94 years, the contraindication between diseases of the oral mucosa and the use of concomitant medication, orthodontic therapy, pregnant, currents oral deleterious habits and surgery, CT or RT current palliative or earlier, besides the adverse effects in a few patients as nausea, vomiting, upper respiratory tract infection and hemoptysis. In their majority, in stage III and IV derived from surgical oncologic treatment and RT with indicating mucositis Gº I or II. The managements of EG and CG vary over their effectivenesses between good and excellent for all indicators of oral complications except for the CG to periodontal infection, being that the poor effectiveness was detonated only in the management of CG for oral mucositis, dysphagia, fungal infections and periodontal. However the suggested managements protocols of OCCROC through the systematic review are limited by small samples of the published studies and the not evidence of the target population of children, adolescents and young. But have low bias risk affirmed by PEDro Scale and associative direction classification, being suitable for dental practice faithfully in the methodology systematically evaluated.

In the meta-analysis with statistical significance for OR, the study of Hadjieva *et al.*, (14) was the one who presented the management of EG with greater therapeutic efficacy and lower risk of development of OMP compared to treatments of other studies (6,13,16,17), being that only the study of Carvalho *et al.*, (16) presented a higher statistical significance in the analysis of RR of management in the GC with therapeutic efficacy lower and higher risk of MDO compared to managements of other studies (6,13,14,17). This meta-analysis is limited to little combination of current studies on the thematic, but it is important for its high quality methodological and low risk of bias for dental clinical application, mainly of the study of Hadjieva *et al.*, (14) with the protocol suggestive for management with CAM2028 (Episil®, Camurus) plus 28,2 mg/mL of benzydamine (nonsteroidal anti-inflammatory) - bioadhesive oromucosal gel for adults with pain coming from the oral mucositis of CT e/or RT of OSCC ( Table 3).

- Implications for future research

The utilization of the protocols suggestive for managements of OCCROC is safe for non-allergic patients to treatment applied according to clinical parameters adopted as from the high methodological quality and low bias risk of the studies systematically reviewed. For future clinical research identified the need to develop study designs with more detailed and rigorous methods, mainly with the triple-blind blinding, representative samples, managements protocols of the OCCROC for children, adolescents and young. Of this form, increasing the number of studies with high methodological a quality and low risk of bias based on the recommendations of the clinical trial checklist for the production of new managements protocols of the OCCROC complete and safe for clinical care in oncology patients.
